# BMI POLYEPIGENETIC SCORES PREDICT FUTURE CHRONIC CONDITIONS AND CHOLESTEROL

**DOI:** 10.1093/geroni/igad104.0723

**Published:** 2023-12-21

**Authors:** Trey Smith, Thomas Karadimas, Helen Meier, Colter Mitchell, Jessica Faul

**Affiliations:** University of Michigan, Ann Arbor, Michigan, United States; University of Michigan, Ann Arbor, Michigan, United States; University of Michigan, Ann Arbor, Michigan, United States; University of Michigan, Ann Arbor, Michigan, United States; University of Michigan, Ann Arbor, Michigan, United States

## Abstract

Body mass index (BMI) is an established risk factor for chronic conditions such as high blood pressure, diabetes, cancer, lung disease, heart condition, stroke, emotional/psychiatric problems, arthritis, and sleep disorders. Poly-Epigenetic Scores (PEGS) have emerged as a promising tool for predicting the risk of chronic diseases. We evaluated the predictive power of BMI PEGS for incident chronic conditions, mortality, and cholesterol levels using data from the Health and Retirement Study, a population representative study of US adults over age 50 years. Three PEGS (Wahl, McCartney, Hamilton) were created from existing epigenome-wide association studies of BMI for participants from the 2016 HRS Venous-Blood study (N=4018). We used element-wise, least-squares regressions to assess the association between each BMI PEG and phenotypes of interest, controlling for demographics, socio-economic status, health-behaviors, and cell type proportions. BMI PEGS were significant predictors of incident chronic conditions in 2020 and these associations were robust to adjustment for covariates. BMI PEGS were also found to be significant predictors of cross-sectional cholesterol levels, a known risk factor for heart disease and stroke. Our study highlights that PEGS can have wide predictive power for future health conditions. These findings can inform personalized interventions for disease prevention and early intervention in the US aging population.

